# History of intraperitoneal platinum drug delivery for ovarian cancer and its future applications

**DOI:** 10.20517/cdr.2020.116

**Published:** 2021-06-19

**Authors:** Franco Muggia, Andrea Bonetti

**Affiliations:** ^1^Department of Medical Oncology, New York University Langone Health, New York, NY 10016, USA.; ^2^Department of Medical Oncology, Mater Salutis Hospital, AULSS 9 of the Veneto Region, Legnago (VR) 37121, Italy.

**Keywords:** Platinum drugs, intraperitoneal administration, epithelial ovarian carcinoma

## Abstract

Intraperitoneal (IP) delivery of cisplatin was developed in the 1970s based on a strong pharmacologic rationale and rodent models. Its advantage over intravenous (IV) administration was supported initially by observational studies in treating recurrent ovarian cancer and eventually by better outcomes from IP *vs.* IV cisplatin in randomized studies in patients undergoing optimal surgical debulking at diagnosis. In the past two decades, with the introduction of novel anticancer interventions (such as taxanes, bevacizumab, inhibitors of DNA repair, and immune check point inhibitors), advantages of IP drug delivery are less clear and concerns are raised on cisplatin's therapeutic index. The discovery of BRCA genes and their key role in DNA repair, on the other hand, have strengthened the rationale for IP drug delivery: high grade serous cancers arising in the Mullerian epithelium in association with hereditary or somatic BRCA function inactivation are linked to peritoneal spread of cells that - while initially sensitive - are prone to emergence of platinum resistance. Therefore, selection of patients based on genomic features and focusing on the better tolerated IP carboplatin are ongoing. Recent examples of leveraging the peritoneal route include (1) targeting the cell membrane copper transport receptor - that is shared by platinums - by the combination of the proteasome inhibitor bortezomib and IP carboplatin; and (2) enhancing IP 5-fluoro-2-deoxyuridine cytotoxicity when coupled with PARP inhibition.

## Introduction

Platinum resistance is the central issue in the treatment of most ovarian cancers. The development of cisplatin as an anticancer agent was reviewed by us at the 50th anniversary of the discovery of its potential anticancer properties^[1]^. Early in its clinical development, cisplatin showed unprecedented activity against ovarian cancer - a disease that is usually diagnosed at advanced stages, with surgery most often playing an ancillary role in its treatment. Although cisplatin - and the subsequent platinum analogs introduced in order to attenuate some of its toxicities - can be relied to induce tumor regressions in up to 90% of late presentations, most women’s cancers eventually recur. In its most common subtype, the high-grade serous adenocarcinoma, tumor progression occurs predominantly in the peritoneal cavity a few months to years after treatment with surgery and the initial “platinum-doublet”. These regimens usually combine a platinum with paclitaxel, with the latter occasionally replaced by other drugs with similar anti-tumor activity as single agent taxanes. Recurrences are commonly classified as “platinum resistant” or “platinum sensitive” based on the timing of when these relapses are documented: if after completing 5 or 6 cycles of platinum-containing regimen, the designation “platinum resistant” is given^[2,3]^. This arbitrary “operational definition” recognizes that cancer reappearance within 6 months of completing 5-6 platinum cycles usually yields at best short-lasting benefits when treated again with a platinum as single agent or as part of a doublet. In fact, even “platinum sensitive” recurrences may reflect some degree of platinum resistance, as suggested by retrospective analysis of 176 women receiving two or more platinum-based regimens for recurrent ovarian cancer, with “the length of a prior response (to the preceding regimen) highly predictive of the upper limit of the duration of response achieved to a subsequent platinum program”^[4]^.

Future investigations need to probe deeper into these definitions and how to deal with consolidation drugs applied after completion of first-line treatment cycles. Insights resulting from the cloning of BRCA genes^[5,6]^, their role in homologous recombination^[7,8]^, the sub-classifications of ovarian cancer based on pathology and genomic characteristics^[9]^, and treatment strategies supplementing chemotherapy are in full development. These advances have further emphasized why optimal utilization of the platinum drugs coupled with knowledge of homologous recombination deficiency (HRD) is central to the treatment of the most common subset of ovarian cancer (also including those serous cancers diagnosed as arising in Fallopian tubes or as primary peritoneal cancers). Typically, such cancers are initially not only highly “platinum sensitive”, presumably from germline or somatic deleterious *BRCA* mutations with HRD, but also characterized by their early peritoneal dissemination leading to peritoneal surface implants. These features, bolstered by clinical experience to be cited in this overview suggest a possible role for intraperitoneal (IP) therapy in controlling these molecular subtypes. After reviewing the historical background for cisplatin administration, we shall comment on the possible integration of IP drug administration in strategies designed to minimize the emergence of platinum resistance. As trialists persuaded by improved IP therapy outcomes^[10]^ - in spite of some justified skepticism^[11]^ - we shall focus on how optimizing platinum pharmacodynamics in selected patients manifesting early spread is the appropriate target for IP platinum administration.

## Intraperitoneal therapy: from historical origins to present trials

In 1964 as a Hematology fellow at Columbia’s Francis Delafield Cancer Hospital (a remarkable 1950s development of the Municipal Hospital system of New York led by its Health Commissioner Ray Trussel), FM recalls treating ovarian cancer patients suffering with massive ascites by injecting ThioTepa directly into their distended abdomens, sometimes repeatedly. Severe thrombocytopenia was dose-limiting, and any improvements observed were short-lived. Such treatment interventions were promptly discarded but remissions after oral melphalan or cyclophosphamide^[12-14]^ led to routine use of these drugs as systemic monotherapy. Cyclophosphamide’s “platelet-sparing” effects led to testing it in drug combinations that eventually claimed to improve median times to progression by a few months over the non-specific DNA damaging alkylating agents^[15]^. At the unusual early stages of ovarian cancer, IP radioactive colloidal chromic phosphate P32 was tested *vs.* melphalan as consolidation in the absence of residual disease after surgery; subsequently, in a randomized trial *vs.* systemic cisplatin + cyclophosphamide with a non-significant advantage in survival for the latter (GOG95)^[16]^.

By the 1970s, the IP route for drug administration had become the focus of pharmacologic and clinical studies at the National Cancer Institute (NCI), National Institutes of Health (NIH). With Vincent T DeVita Jr, assuming the Directorship of the Division of Cancer Treatment in 1974 to succeed C Gordon Zubrod - who in 1954 was recruited to run the Chemotherapy Program of NCI emphasizing anticancer drug development for hematologic malignancies - its Medicine Branch turned its attention to curative strategies for breast and ovarian cancers. Laparoscopic staging and drug combinations were the initial focus of ovarian cancer clinical protocols. Out of these studies, led by Robert C Young established benefits from the regimen HexaCAF (hexamethylmelamine, cyclophosphamide, methotrexate/Amethopterin and 5-fluorouracil, soon to be eclipsed by the emergence of cisplatin. Its chemical structure was identified by Rosenberg’s laboratory observations at Michigan State and thereafter its development relied on the NCI for preclinical and clinical development)^[1,17]^. Medicine Branch trainees (e.g, Robert Ozols, William McGuire, Charles Myers, and James Speyer) became involved in this area of therapeutics and the first two started extensive interactions with extramural gynecologists who since 1970 with NCI support began clinical trials under the umbrella of the Gynecologic Oncology Group (GOG)-paralleling actions created under NCI Clinical Cooperative Groups seeking to integrate clinical drug development with surgery and radiation. GOG became the centerpiece for the future testing of cisplatin and paclitaxel - the most successful anticancer drugs emerging from studies fully coordinated by the NCI.

The pharmacologic basis for IP drug delivery was developed by Robert L Dedrick at the NIH^[18]^, and soon thereafter a number of phase I/II and pharmacokinetics studies with methotrexate, 5-fluoruracil, and the novel antitumor antibiotic doxorubicin were launched at the Medicine and Surgery Branches under DeVita’s prompting^[19-21]^. Initially, the clinical focus was on drug peritoneal/plasma concentration ratios that were tolerable, but drug diffusivity (penetrance) into tissues and intrinsic tumor sensitivity to a drug was later emphasized as being most relevant to eventual clinical results^[22]^. In fact, Stephen Howell at University of California, San Diego, initiated IP cisplatin studies after the 1979 approval for systemic treatment of ovarian cancer, and reported the most noteworthy clinical results^[23]^. These were compelling enough for Baltimore NCI unit-trained David Alberts in 1985 to a phase III study of cisplatin IP *vs.* IV (+ IV cyclophosphamide in both arms) within the Ovarian Committee of the Southwest Oncology Group (SWOG). This study completed its lagging accrual with GOG participation (GOG104): its results presented at the American Society of Clinical Oncology plenary session and published in 1996 fostered enthusiasm for further exploration of IP cisplatin^[24]^. Accordingly, it was followed by GOG114^[25]^: this study adopted the new standard IV cisplatin + 24h IV paclitaxel as a comparator to the IP cisplatin arm. However, this experimental IP arm was modified to be preceded by IV carboplatin AUC 9 × 2 cycles as “chemical debulking” before IP cisplatin + 24h IV paclitaxel; this resulted in excessive hematologic toxicity. Therefore, even with an improved overall median survival, because of its greater toxicity it could not be “recommend for routine use”. Such a conclusion led to the third GOG IV *vs.* IP comparison in GOG172^[26]^ that included yet another major change in the experimental IP regimen, - now adding a day 8 IP paclitaxel to the IP cisplatin. The median survival results exceeding 5 years first disclosed at the GOG meeting were striking for the IP arm, even though associated with considerably more toxicity and lesser completion rates. An NCI Clinical Announcement followed in January 2006 urging IP treatments was provided for optimally cytoreduced women with stage III ovarian cancer. With each of the IP arms differing among the 3 GOG studies and problematic toxicities blunted adoption of IP regimens outside the US. The late Martin Gore articulated the European position over several years with insightful comments: e.g, “even if IP superiority is confirmed by a subsequent trial, tell me how I should use it”^[27]^, and after the brilliant results of GOG172^[26]^, his overall critique was “Lack of robustness of the data lies in the design of the main IP trials, not their conduct or analysis”. Introduction of IP paclitaxel in GOG172, in particular, was problematic for testing the original hypothesis of IP *vs.* IV cisplatin and it raised new local and systemic toxicity issues (especially neurotoxicity). Additionally, critics of IP approaches objected that the comparator in the IV arm was cisplatin and not the by now current standard IV carboplatin (based on Ozols’ non-inferiority trial GOG158 comparing the two platinums, both with IV paclitaxel, that favored carboplatin)^[28]^.

In the GOG172 IP regimen the long-lasting neurotoxicity and other concerns associated with cisplatin for up to 6 cycles at the excessive doses (for women) of 100 mg/m^2^ given on day 2 (coupled with IV paclitaxel on day 1) were further compounded by an additional paclitaxel IP on day 8. Therefore, the GOG embarked on piloting several modified IP regimens with cisplatin at 75 mg/m^2^ (but still retaining the IP paclitaxel on day 8). After 3 years of IP pilot studies, other concepts gained traction (weekly-dose dense-paclitaxel based on Japanese GOG, and bevacizumab impact in GOG and ICON groups). These innovations led to GOG252: a 3-way comparison enrolling 1,560 women randomized to (1) a reduced cisplatin dose-modified GOG172 IP regimen; (2) 80 mg/m^2^ IV paclitaxel d1, 8, 15 (acquired from the Japanese GOG study) given together with IP carboplatin at target AUC dosing of 6 or 3) the same two drugs and respective doses with both given IV. Bevacizumab every 3 weeks was added to each arm^[29]^. The final analysis of this study was published in 2019 - three years after initial analysis of progression free survival (PFS) had been presented showing no significant differences between the 3 arms but with quality of life less affected in the IP or IV carboplatin arms. As anticipated, the mature overall survival (OS) analysis was similar in all three arms, exceeding 6 years in all three regimens. These results were definitive enough to reverse any impact on clinical practice including IP cisplatin administration that the 2006 Clinical Announcement had recommended. The brisk enrollment into this trial, however, reflects the ease gynecologic oncologists have adopted IP interventions, not impeding entry into such administration into studies. The overall lack of advantage in outcome, however, has essentially nullified IP interventions as a standard option relative to IV regimens that uniformly rely on carboplatin doublets. Whether differences were blurred by introducing weekly paclitaxel in two of the arms (and also on d8 IP in the modified GOG172 regimen) and by bevacizumab improving PFS in all three arms, or by diminished efficacy of the lower IP cisplatin dose (75 mg/m^2^ per cycle rather 100 g/m^2^) is a currently unanswerable question. While trialists consider future directions of IP interventions, the Dutch group randomized patients to hyperthermic IP chemotherapy (HIPEC) utilizing cisplatin × 1 HIPEC following 3 cycles of carboplatin/paclitaxel and surgical cytoreduction *vs.* the same surgical intervention without HIPEC. Both arms went on to receive additional 3 cycles of carboplatin/paclitaxel. This study provided a statistically significant survival advantage for the HIPEC arm^[30]^. HIPEC has been applied at different time points of treatment of advanced epithelial ovarian carcinoma, namely up-front, interval, and recurrent setting^[31]^. However, the use of HIPEC following neo-adjuvant chemotherapy (NACT) at the time of interval debulking surgery is the most promising since it is based on a higher level of evidence^[30]^ and perhaps should be able to reverse the platinum-resistance induced by neo-adjuvant chemotherapy^[32]^. Furthermore, the observation that hyperthermia delayed the repair of DNA damage induced by cisplatin or doxorubicin acting upstream of different repair pathways to block histone polyADP-ribosylation (PARylation), as efficiently as pharmacologic inhibitors of PARP (PARPi), producing comparable delay in DNA repair, induction of double-strand breaks, and cell cytotoxicity after chemotherapy could suggest a potential synergistic effect of HIPEC and PARP1 inhibitor^[33]^, to be evaluated in clinical trials. In the post-NACT and optimal surgical debulking setting the Gynecologic Cancer Intergroup performed a two-stage adaptively designed randomized phase II study of intraperitoneal *vs.* intravenous chemotherapy^[34]^. Patients were randomized to one of 3 arms: (1) i.v. carboplatin/paclitaxel control arm; (2) i.p. cisplatin plus i.v./i.p. paclitaxel; or (3) i.p. carboplatin plus i.v./i.p. paclitaxel with the primary endpoint being 9-month progressive disease (PD9) rate before going on to an anticipated two-arm phase III comparison of the intravenous standard *vs.* one of the intraperitoneal arms. The IP cisplatin arm did not progress beyond the first stage of the study after failing to meet the PD9 pre-set superiority rule the final analysis compared i.v. carboplatin/paclitaxel (*n* = 101) while i.p carboplatin, i.v./i.p. paclitaxel (*n* = 102) did show a lower PD9 rate compared with the i.v. carboplatin/paclitaxel control arm: 24.5% (95%CI: 16.2%-32.9%) *vs.* 38.6% (95%CI: 29.1%-48.1%) (*P* = 0.065). Regrettably, the planned phase III trial was aborted due to insufficient funding.

## IP platinum-based therapy: refining the questions

Most positive signals from IP drug administration have been obtained in epithelial ovarian cancer with high-grade serous cancers and early peritoneal spread - many originate in the fimbria of the Fallopian tubes^[35-37]^. Early NCI studies on IP therapy concentrated on ovarian cancer because one could attain a pharmacologic advantage with a number of chemotherapy agents. Subsequently, the IP route would be deemed ideal following “early diagnosis” or “optimally debulked” in a disease initially confined to the peritoneal cavity -for example, we have since learned from BRCA mutation-related serous cancers discovered incidentally at risk-reducing salpingo-oophorectomies in hereditary mutation carriers. The studies conducted by UCSD and the GOG reviewed in detail had signaled improvements in outcome for IP cisplatin administration and reinforced by the initial 3 GOG studies utilizing cisplatin at 100 mg/m^2^ per cycle, but varied in some aspects of patient eligibility (tumor residual) as well as accompanying added treatments in the IP cisplatin arm. In the last GOG252, all differences became blurred with superior overall outcome results suggesting a contribution of bevacizumab to IV and both IP arms. Rather than discarding the prior undoubtedly positive signals following IP drug administration, we suggest that one should consider the following steps: (1) refining eligibility to concentrate on HRD deficient disease and where most favorable low-volume disease distribution might be expected; (2) testing out other potentiators of IP platinums; and (3) reinforcing potential practical advantages of IP administered drugs.

## IP route: patient selection

As a reference point, the NCI The Cancer Genome Atlas on ovarian cancer provided a comprehensive analysis of genomic alteration in high grade serous cancers^[9]^. If an advantage from IP administration over IV is going to emerge, the patient selection should initially be confined to those with demonstrable genetic mutations of BRCA and/or other germline or somatic alterations resulting in HRD. Retrospective studies have shown an improved prognosis, higher response rates to platinum-containing regimens, and longer treatment-free intervals between relapses in patients with BRCA 1 and BRCA 2 (BRCA1/2)-mutated ovarian cancer compared with patients who are not carriers of this mutation^[38]^. Regarding the role of IP therapy in BRCA-mutated ovarian cancer, an ancillary study of a subset of GOG 172 patients revealed that those with reduced *BRCA 1* expression greatly benefitted from IP rather than IV chemotherapy: in tumors with aberrant *BRCA1* expression the median OS was 84 months *vs.* 47 months in the IP *vs.* IV group, respectively (*P* = 0.0002)^[39]^. Moreover, in a retrospective study of OS among 62 patients entering in IP platinum doublet phase II trials performed at NYU, a subset of 10 patients later documented to have germline BRCA mutations experienced a very long median survivals (10+ years)^[40]^.

Beyond BRCA mutations and other HRD features, patients should be optimally cytoreduced up front and include only stages II or III. Anyone undergoing additional resections of bowel and visceral organs might also need to be excluded. To avoid additional issues not related to IP *vs.* IV comparisons, one should consider using the same platinum agent in IV and IP arms, that is, carboplatin. In this era of pretreatment genomic analysis, one could include additional accompanying biomarker studies of outcome, and detection of early recurrence by ctDNA, as an example. In addition to attempting to confirm an IP *vs.* IV advantage in this selected population, the IP delivery lends itself to potentiating the action of platinums by this route. The next section will describe studies that provided positive leads but unfortunately have not followed up to date, in part because clinical investigations without pharmaceutical industry sponsorship are challenging to get underway more than ever.

## IP route: addressing new hypotheses in overcoming platinum resistance using IP therapy

Data from Howell’s laboratory targeting the cell membrane copper transport receptor (CTR) shared by platinums suggested that cisplatin resistance was overcome by the combination of the proteasome inhibitor bortezomib and cisplatin^[41,42]^. The GOG set out to prove this hypothesis by conducting a clinical trial in patients with peritoneal recurrence mimicking Howell’s preclinical data. In the study by Jandial *et al*.^[43]^, 33 heavily pretreated women meeting eligibility criteria were enrolled in the phase I/pharmacokinetic study to evaluate the safety of IP carboplatin AUC 5 and bortezomib 0.5 mg/m^2^. Twenty-one of these patients had measurable disease with one complete and 3 partial responses (overall response rate 19%) were noted. With the clinical experience of IP placement over several decades the leads from this phase I study should be pursued among other hypotheses that need to be tested about overcoming resistance, especially since this study demonstrated clinical benefit with a satisfactory therapeutic index. Moreover, it exemplifies a path of going from animal models into human study that has not been exploited.

Series of trials utilizing the IP route in patients with ovarian cancer recurrences to assess potential drug activity alone and in combination with platinums were carried out by the GOG102 in the early 1990s (terminated when joining the phase III SWOG study as GOG104). We had studied IP fluoropyrimidines, and specifically 5-fluoro-2-deoxyuridine (FUDR) as single agent^[44]^ and this arm was one of the two selected of a SWOG randomized phase 2 study^[45]^. In this trial its progression free survival vastly exceeded that of IP mitoxantrone that had been selected based on anecdotal reports of activity. Subsequently, we went on to combine FUDR with platinums for further development as a promising combination but it was displaced by the emerging interest of IP paclitaxel (that was later included in GOG172 and in the modified arm of GOG252). While further development stalled, Scott Kaufmann’s laboratory studies in Mayo Clinic had established that IP FUDR but not 5-fluorouracil potentiated the effect of PARP inhibitors^[46,47]^. This led to an institutional Phase I clinical study of IP FUDR coupled with oral veliparib (A. Wahner Hendrickson, unpublished) that completed accrual in 2019. These are examples of how leveraging PARP inhibition and IP drug combinations overcome relative resistance to the platinum drugs that may be less achievable by the systemic route. All our experience with peritoneal ports may allow monitoring for ctDNA in peritoneal washings. In fact, in our unpublished experience based on monitoring peritoneal cytology every 6 weeks × 4 after completion of IP treatment does lead to a 10% early detection of peritoneal recurrences not otherwise detectable by imaging or CA125 increase (FM, personal communication). Removing the port at 6 months was chosen as a compromise of having to deal with the port a few more months after completion of treatment (unless chronically uncomfortable) and detecting a “platinum-resistant” peritoneal relapse that might be appropriate for experimental IP therapies.

## Overview and future directions

Platinum-resistance is a key element of the challenging relapses that follows all approaches to high-grade serous ovarian cancers. We have reviewed how intraperitoneal therapy was developed and its pharmacologic basis led to phase III trials that have provided mostly positive signals, but also associated with increasing toxicities. A summary of published phase III trials comparing IP with IV administrations of platinum-containing regimens, including studies performed before the introduction of taxanes^[48-52]^ is reported in Table 1. The table is built upon the paper published by the Cochrane library^[53]^ and by the Cancer Care Ontario^[54]^. The Cochrane concluded that women receiving primary treatment for ovarian cancer were less likely to die if they received an IP component to chemotherapy (eight studies, 2026 women; HR = 0.81; 95%CI: 0.72 to 0.90) and that there was greater serious toxicity with regard to gastrointestinal effects, pain, fever and infection but less ototoxicity with the IP than the IV route. Table 2 summarizes potential advantages and disadvantages of the IP route as research strategy vis-à-vis the IV (standard) route. Finally, the GOG 252 trial demonstrates IP drug delivery may be integrated into clinical practice with relative ease. Our historical overview provides a background whereby new studies may address key questions in overcoming platinum resistance utilizing the IP route, addressing questions by collaborating with basic and translational scientists.

**Table 1:** Intraperitoneal *vs.* Intravenous Phase III trials

Study	No. of patients	IP *vs.* Control	Chemotherapy	Median PFS (months)	*P* value	Median OS (months)	*P* value
Zylberberg *et al.*^[48]^ 1986	20	IV/IP Control	Arm 1: IV adriamycin 35 mg × 2 + fluorouracil 750 mg × 2 + bleomycin 15 mg + cisplatin 100 mg + vincaleucoblastine 10 mg + ifosfamide 1 g × 2 Arm 2: IV adriamycin 20 mg × 2 + fluorouracil 500 mg × 2 + cisplatin 50 mg + vincaleucoblastine 10 mg + ifosfamide 1 g × 2 + IP bleomycin 15 mg + cisplatin 50 mg + fluorouracil 500 mg + adriamycin 30 mg	NR		NR	
Kirmani *et al.*^[49]^ 1994	29 33	IP Control	IP Cisplatin 200 mg/m^2^ + IP Etoposide 350 mg/m^2^ every 28 days for 6 cycles IV cisplatin 100 mg/m^2^ + IV cyclophosphamide 600 mg/m^2^ every 21 days for six cycles	14 12	0.46	32 36	0.45
GOG 104 1996^[24]^	267 279	IP Control	IV cyclophosphamide 600 mg/m^2^ + IP cisplatin 100 mg/m^2^ IV cyclophosphamide 600 mg/m^2^ + IV cisplatin 100 mg/m^2^	NR NR		49 41	0.02
Polyzos *et al.*^[50]^ 1999	44 46	IP Control	IP Carboplatin 350 mg/m^2^ + IV cyclophosphamide 600 mg/m^2^ every three weeks for 6 cycles IV Carboplatin 350 mg/m^2^ + IV cyclophosphamide 600 mg/m^2^ every three weeks for 6 cycles	18 19	NS	26 25	NS
Gadducci *et al.*^[51]^ 2000	113	IP Control	IP cisplatin 50 mg/m^2^ + IV doxorubicin 60 mg/m^2^ + IV cyclophosphamide 600 mg/m^2^ every 4 weeks for 6 cycles IV cisplatin 50 mg/m^2^ + IV doxorubicin 60 mg/m^2^ + IV cyclophosphamide 600 mg/m^2^ every 4 weeks for 6 cycles	42 25	0.13	67 51	0.14
Yen *et al.*^[52]^ 2001	55 63	IP Control	IP cisplatin 100 mg/m^2^ + IV cyclophosphamide 500 mg/m^2^ every three weeks for 6 cycles IP cisplatin 50 mg/m^2^ + IV cyclophosphamide 500 mg/m^2^ every three weeks for 6 cycles	NR		43 48	0.47
GOG 114 2001^[25]^	235 227	IP Control	IV carboplatin AUC 9 every 28 days for two cycles, followed 28 days later by IV paclitaxel 135 mg/m^2^ over 24 h (day 1) + IP cisplatin 100 mg/m^2^ (day 2) IV paclitaxel 135 mg/m^2^ over 24 h (day 1) + IV cisplatin 75 mg/m2 (day 2)	28 22	0.01	63 52	0.05
GOG 172 2006^[26]^	205 210	IP Control	IV paclitaxel 135 mg/m^2^ over 24 h (day 1) + IP cisplatin 100 mg/m^2^ (day 2) + IP paclitaxel 60 mg/m^2^ (day 8) IV paclitaxel 135 mg/m^2^ over 24 h (day 1) + IV cisplatin 75 mg/m^2^ (day 2)	24 18	0.05	65.6 49.7	0.03
GOG 252 2019^[29]^	518 521 521	IP carbo IP cis Control	IV paclitaxel 80 mg/m2 weekly + IP carboplatin AUC 6 + bevacizumab 15 mg/kg every 3 weeks IV paclitaxel 135 mg/m^2^ over 3 h day 1 + IP cisplatin 75 mg/m^2^ day 2 + IP paclitaxel 60 mg/m^2^ day 8 + bevacizumab 15 mg/kg every 3 weeks IV paclitaxel 80 mg/m^2^ weekly + IV carboplatin AUC 6 + bevacizumab 15 mg/kg every 3 weeks (all arms bevacizumab from cycles 2-22)	27.4 26.2 24.9	N.S.	78.9 72.9 5.5	NS

*This is the earliest RCT on IP chemotherapy for the initial management of epithelial ovarian cancer. The Authors described a statistically significant increase in the number of women alive and free of disease in the IP group (*P* < 0.05) but no further statistics were provided^[47]^.

**Table 2:** Advantages and disadvantages of intraperitoneal drug administration

Advantages	Disadvantages
↑↑Drug concentration at peritoneal disease sites	May not apply to sizable tumors & areas of poor exposure
Mostly “active” cisplatin after IP delivery in NS	Ongoing cisplatin inactivation after IV delivery
FUdR mostly intact at peritoneal disease sites	Not feasible IV: FUdR IV is hydrolyzed to 5-fluorouracil (5FU) & no synergy with PARPi
Synergy of IP FUdR with oral PARP inhibitors	FUDR requires port to synergize PARPi
IP Port may facilitate cytological sampling	Surgery to insert access port; malfunction ~ 10%
IP drugs enter circulation: reach tumors also by capillary flow, but less peak levels (for cisplatin that translates in lesser nephro- and oto-toxicity	Drug exposure of tumors beyond surface could be less than after IV administration

NS: Normal saline; FUdR: 5-fluoro-2-deoxyruridine; 5FU: 5-fluoruracil; PARPi: inhibitors of poly (ADP-ribose) polymerase.

## Declarations

### Copyright

© The Author(s) 2021.
